# Factors Associated with Inadequate Birth Intervals in the BRISA Birth Cohort, Brazil

**DOI:** 10.1055/s-0040-1701463

**Published:** 2020-02

**Authors:** Raphael Barbosa, Maria Teresa Seabra Soares Britto Alves, Ian Nathasje, Deysianne Chagas, Vanda Ferreira Simões, Leonardo Silva

**Affiliations:** 1 Department of Public Health, Universidade Federal do Maranhão, São Luís, MA, Brazil

**Keywords:** birth interval, birth, women's health, family planning, reproductive health, intervalo de nascimento, nascimento, saúde da mulher, planejamento familiar, saúde reprodutiva

## Abstract

**Objective** To determine the prevalence of inadequate birth interval and its associated factors in the BRISA study.

**Methods** Cross-sectional study using data from the BRISA cohort. Birth interval was categorized into “adequate” (≥ 2 years or < 5 years between births), “short interval” (< 2 years) and “long interval” (≥ 5 years). The analysis of the factors associated with short and long birth intervals used multinomial logistic regression.

**Results** The prevalence of adequate birth intervals was 48.3%, of long intervals, 34.6%, and of short intervals, 17.1%. Skin color, age, education level, economic status, type of delivery, number of prenatal visits, parity, blood pressure, diabetes, and anemia (*p*-value was < 0.2 in the univariate analysis) proceeded to the final model. The variable ≥ 3 births (odds ratio [OR] = 1.29; confidence interval [CI]: 1.01–1.65) was associated with short intervals. Age < 20 years old (OR = 0.48; CI: 0.02–0.12) or ≥ 35 years old (OR = 2.43; CI: 1.82–3.25), ≥ 6 prenatal visits (OR = 0.58; CI: 0.47–0.72), ≥ 3 births (OR = 0.59; CI: 0.49–0.73), and gestational diabetes (OR = 0.38; CI: 0.20–0.75) were associated with long intervals.

**Conclusion** Older mothers were more likely to have long birth intervals, and higher parity increases the chances of short birth intervals. Furthermore, gestational diabetes and adequate prenatal care presented higher chances of having adequate birth intervals, indicating that health assistance during pregnancy is important to encourage an adequate interval between gestations.

## Resumo

**Objetivo** Determinar a prevalência de intervalo inadequado de nascimento e seus fatores associados no estudo BRISA.

**Métodos** Estudo transversal com dados da coorte BRISA. O intervalo de nascimento foi categorizado em “adequado” (≥ 2 anos ou < 5 anos entre os nascimentos), “intervalo curto” (< 2 anos) e “intervalo longo” (≥ 5 anos). A análise dos fatores associados aos intervalos de nascimento curtos e longos utilizou regressão logística multinomial.

**Resultados** A prevalência de intervalos adequados de nascimento foi de 48,3%, longa de 34,6% e curta de 17,1%. A cor da pele, idade, escolaridade, status econômico, tipo de parto, número de consultas pré-natais, paridade, pressão arterial, diabetes e anemia (valor-*p* < 0,2 na análise univariada) prosseguiram para o modelo final. A variável ≥ 3 nascimentos (odds ratio [OR] = 1,29; intervalo de confiança [IC]: 1,01–1,65) esteve associada a intervalos curtos. Idade < 20 anos (OR = 0.48; CI: 0.02–0.12) ou ≥ 35 anos (OR = 2.43; CI: 1.82–3.25), ≥ 6 consultas pré-natais (OR = 0.58; CI: 0.47–0.72), ≥ 3 nascimentos (OR = 0.59; CI: 0.49–0.73), e diabetes gestacional (OR = 0.38; CI: 0.20–0.75) foram associados a longos intervalos.

**Conclusão** As mães mais velhas apresentaram maior probabilidade de ter longos intervalos de nascimento, e uma paridade maior aumenta as chances de intervalos curtos de nascimento. Além disso, o diabetes gestacional e o pré-natal adequado apresentaram maiores chances de ter um intervalo adequado ao nascimento, indicando que a assistência à saúde durante a gravidez é importante para incentivar um intervalo adequado entre as gestações.

## Introduction

The World Health Organization (WHO) recommends waiting between 18 to 24 months, but < 5 years, after a live birth before attempting another pregnancy.^1^ ^2^ Birth intervals < 24 months are considered short and intervals > 5 years are considered long.^1^ ^2^ ^3^ ^4^ ^5^ Both short and long intervals are considered inadequate. This recommendation aims to reduce the risk of adverse maternal, perinatal, and infant health outcomes and is consistent with the WHO/UNICEF recommendation that breastfeeding should be maintained for at least 2 years.^2^ ^6^

Research has shown that long and short interpregnancy intervals are independently associated with an elevated risk of adverse maternal, perinatal, and infant health outcomes.^3^ Adverse maternal health outcomes include anemia, gestational hypertension, and maternal death.^4^ ^5^ ^6^ ^7^

Literature on this topic has tended to focus on the consequences of short and long birth intervals for perinatal outcomes. The effects of birth spacing on maternal mortality and morbidity have received less attention. Therefore, relatively little is known about its consequences and associated factors.^2^ ^6^ ^8^ ^9^ ^10^ ^11^

Short interpregnancy intervals are associated with increased adverse maternal health outcomes, such as risk of premature rupturing of membranes, preterm birth, uterine and placental bleeding, and gestational diabetes,^3^ ^6^ ^8^ ^9^ ^10^ ^12^ while long intervals can increase the risk of gestational hypertension or preeclampsia.^7^ ^9^ ^10^ ^11^ ^12^ ^13^

The length of birth interval is influenced by socioeconomic, demographic, and reproductive health factors. In this respect, studies have shown that factors related to short intervals include low socioeconomic status, postpartum stress, unstable lifestyles, and access to health services, while advanced maternal age, maternal illness, infertility, unplanned pregnancy, and family and social break-ups are potential factors associated with long intervals. These factors can influence maternal health independently of their effect on birth interval.^6^ ^8^ ^9^ ^10^

Studies have also investigated the influence of prenatal care on maternal and reproductive health and, by association, the relationship between this factor and birth interval.^13^ ^14^ ^15^ The aim of prenatal care, besides providing adequate assistance during pregnancy, is to promote maternal, family, and infant health.^13^ ^14^ ^15^ ^16^ ^17^ Prenatal care can also facilitate fertility planning because it represents an opportunity for pregnant women to keep in contact with health and social services. However, the influence of this factor on future reproductive behavior remains unclear.^13^ ^14^

Among women in Africa, inadequate birth spacing was rated as more risky for women's health than other pregnancy-related events, like contraceptive methods.^18^

In view of the above, it is essential to gain a deeper understanding of the factors influencing birth intervals and the association between these intervals and maternal morbidity. The aim of the present study was, therefore, to determine birth intervals and to investigate the socioeconomic and reproductive health factors and maternal morbidities associated with inadequate birth intervals in São Luís, state of Maranhão, in the northeastern region of Brazil.

## Methods

A cross-sectional population-based study was conducted using data on hospital births in São Luís from the BRISA birth cohort. The present study was conducted in 10 public and private hospitals and maternity facilities in the municipality of São Luís using a representative sample (one-third) of births in these facilities in 2010. The total sample comprised 5,067 births after the exclusion of stillbirths and twins.^19^

A stratified sampling design was used, in which the size of each stratum was proportional to the number of births in each maternity facility. The births from each maternity facility were selected systematically from a list of all births that occurred in the facility sorted in chronological order, using a sampling interval of three and a random starting point between one and three. Starting with the random starting point, we counted down the list selecting each third birth until the desired number of births was selected.^19^

Data was collected using a standardized questionnaire answered by mothers, preferably in the first 24 hours after birth. Information on prenatal care was obtained from verbal reports of the mothers or from maternity records, when available.

The questionnaire was divided into 11 blocks. Blocks C and G contained questions about socioeconomic status and demographic characteristics, and maternal morbidities during pregnancy, respectively, while block H included questions about the current pregnancy and prenatal care.

The following variables were analyzed: birth interval, skin color, age, economic status, education level, type of delivery, number of deliveries, number of prenatal visits, high blood pressure, diabetes, bleeding in the 3^rd^ trimester, and anemia.

Birth interval was calculated by using the difference between the current age of the mother and her age in the last pregnancy. The data were self-reported and were categorized into “adequate” (≥ 2 years or < 5 years between births), “short interval” (< 2 years), and “long interval” (≥ 5 years) based on recommendations proposed by the Report of a WHO Technical Consultation on Birth Spacing.^1^ Skin color was self-reported and categorized into white (participants who responded “white”) and nonwhite (those who responded “black,” “brown/mulatto/*cabocla*/*morena*,” “yellow/oriental,” or “indigenous”). Age was divided into three categories: < 20 years old, 20 to 34 years old, and ≥ 35 years old. Economic status was classified according to the Brazilian Criteria of Economic Classification into groups A, B, C, D, and E^17^ ^20^ and regrouped into three categories: A-B, C, and D-E. Education level was assessed based on years of study and divided into two categories: < 9 years and > 9 years.

With regard to reproductive health variables, delivery type was classified as natural birth and cesarean section, while number of deliveries was grouped into 2 deliveries and ≥ 3 deliveries. The number of prenatal visits was defined according to the minimum number recommended by the Brazilian Ministry of Health^20^ and was categorized into < 6 and > 6 visits. Maternal morbidities, such as high blood pressure, diabetes, anemia, and bleeding in the 3^rd^ trimester, were assessed in relation to the current pregnancy.

For the present article, the minimum sample size was determined based on an expected prevalence of 50%, precision of 2%, and 95% confidence interval (CI), resulting in 2,396 women. From the total sample of the BRISA study, all women who reported more than one delivery, live birth, and singleton pregnancies were included in this analysis.

Data analysis was conducted using the statistical software package Stata 14.0 (Statacorp, College Station, TX, USA). A descriptive analysis of the data was conducted to determine the frequencies and percentages of the variables. The analysis of the factors associated with short and long birth intervals used multinomial logistic regression, using the adequate interval as the reference category.

Univariate analysis was conducted first to determine unadjusted odds ratios (ORs) adopting a 95% CI. The independent variables that obtained a p-value < 0.20 were included in the multivariate analysis. A significance level of 5% was adopted.

In accordance with the provisions of Resolution 196/96 of the National Health Council, the present study was approved by the Ethics Committee of the Hospital Universitário Presidente Dutra (application number 4771/2008–30 and 223/2009).

## Results

**Fig. 1:**
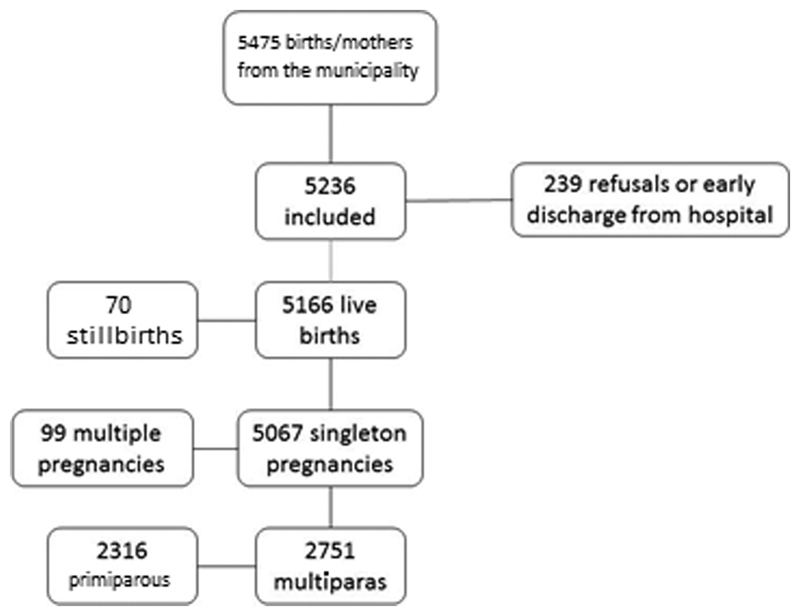
Flowchart of the selection of eligible participants and final sample of the BRISA study.

The final sample was comprised of 2,751 mothers who gave birth in maternity facilities in São Luís, state of Maranhão, Brazil (Fig. 1). The prevalence of adequate birth intervals (48.3%) was higher than that of long (34.6%) and short (17.1%) intervals (Table 1).

**Table 1:** Women's birth interval in the BRISA study

Birth interval	*n* (%)
**Adequate interval**	1,328 (48.3)
**Short interval**	471 (17.1)
**Long Interval**	952 (34.6)

The majority of the women were nonwhite, from economic group C, aged between 20 and 24 years old, had at least 9 years of education, had 2 lifetime births, had vaginal births, and had at least 6 prenatal care visits; 15.6% of the respondents had high blood pressure during pregnancy, 2.2% had diabetes, 49.5% had anemia, and 9.4% had had at least one bleeding episode in the 3^rd^ trimester of pregnancy (Table 2).

**Table 2:** Socioeconomic and reproductive health characteristics and maternal morbidity during the last pregnancy

Variables	Adequate interval	Short interval	Long interval	Total
	*n* (%)	*n* (%)	*n* (%)	*n* (%)
Skin color				
White	223 (16.8)	62 (13.2)	181 (19.0)	466 (17.0)
Nonwhite	1,103 (83.2)	409 (86.8)	770 (81.0)	2,282 (83.0)
Age (years old)				
< 20	154 (11.6)	42 (8.9)	6 (0.6)	202 (7,3)
20–34	1,078 (81.2)	391 (83.0)	765 (80.4)	2,234 (81.2)
≥ 35	96 (7.2)	38 (8.1)	181 (19.0)	315 (11.5)
Education level (years)				
< 9	476 (36.1)	192 (41.3)	268 (28.3)	936 (34.3)
≥ 9	842 (63.9)	273 (58.7)	678 (71.7)	1,793 (65.7)
Economic status				
A – B	180 (14.3)	57 (13.6)	193 (21.4)	430 (16.6)
C	617 (49.0)	209 (49.8)	506 (56.0)	1,332 (51.6)
D – E	462 (36.7)	154 (36.7)	204 (22.6)	820 (31.8)
Type of delivery				
Normal	816 (61.4)	284 (60.3)	491 (51.6)	1,591 (57.8)
Cesarean section	512 (38.6)	187 (39.7)	461 (48.4)	1,160 (42.2)
Number of prenatal visits				
< 6	395 (30.2)	116 (25.4)	458 (49.2)	969 (36.0)
≥ 6	913 (69.8)	341 (74.6)	472 (50.8)	1,726 (64.0)
Number of births				
2	751 (56.6)	229 (48.6)	625 (65.7)	1,605 (58.3)
≥ 3	577 (43.4)	242 (51.4)	327 (34.3)	1,146 (41.7)
High blood pressure				
Yes	195 (14.7)	69 (14.7)	166 (17.4)	430 (15.6)
No	1,132 (85.3)	402 (85.3)	786 (82.6)	2,320 (84.4)
Diabetes				
Yes	13 (1.0)	11 (2.3)	37 (3.9)	61 (2.2)
No	1,312 (99.0)	460 (97.7)	915 (96.1)	2,687 (97.8)
Bleeding in 3rd trimester				
Yes	118 (8.9)	46 (9.8)	94 (9.9)	258 (9.4)
No	1,209 (91.1)	425 (90.2)	857 (90.1)	2,491 (90.6)
Anemia				
Yes	671 (50.8)	41 (51.3)	446 (46.9)	1,358 (49.5)
No	650 (49.2)	229 (48.7)	505 (53.1)	1,384 (50.5)

In the univariate analysis for short birth intervals, a significant association was found with ≥ 3 deliveries (OR = 1.38; 95%CI 1.11–1.70). For long intervals, a significant association was observed with age < 26 years old (OR = 0.05; 95%CI: 0.02–0.12) or ≥ 35 years old (OR = 2.66; 95%CI: 2.04–3.46); > 9 years of education (OR = 1.43; 95%CI: 1.19–1.71); economic status C (OR = 0.76; 95%CI: 0.60–0.98) or D/E (OR = 0.41; 95%CI: 0.32–0.53); cesarean section (OR = 1.50; 95%CI: 1.26–1.77); ≥ 6 prenatal visits (OR 0.45; 95%CI: 0.37–0.53); ≥ 3 births (OR 0.68; CI95%: 0.57–0.81); and gestational diabetes (OR = 0.25; 95%CI: 0.13–0.46). No significant association was found between the other variables studied (Table 3).

**Table 3:** Univariate analysis of short and long birth intervals, socioeconomic and reproductive health variables, and maternal morbidity

Variables	Short interval*	Long interval*	*p-value*
	OR (95%CI)	OR (95%CI)	
Skin color			0.02
White	Ref.	Ref.	
Nonwhite	1.33 (0.98–1.80)	0.86 (0,69–1,06)	
Age (years old)			< 0.01
< 20	0.75 (0.52–1.08)	0.05 (0.02–0.12)	
20–34	Ref.	Ref.	
≥ 35	1.09 (0.74–1.62)	2.66 (2.04–3.46)	
Education level (years)			
< 9	Ref.	Ref.	< 0.01
≥ 9	0.80 (0.65–1.00)	1.43 (1.19–1.71)	
Economic status			< 0.01
A–B	Ref.	Ref.	
C	1.07 (0.76–1.50)	0.76 (0.60–0.98)	
D–E	1.05 (0.74–1.49)	0.41 (0.32–0.53)	
Type of delivery			< 0.01
Normal	Ref.	Ref.	
Cesarean section	1.05 (0.85–1.30)	1.50 (1.26–1.77)	
Number of prenatal visits			< 0.01
< 6	Ref.	Ref.	
≥ 6	1.27 (1.00–1.62)	0.45 (0.37–0.53)	
Number of births			< 0.01
2	Ref.	Ref.	
≥ 3	1.38 (1.11–1.70)	0.68 (0.57–0.81)	
High blood pressure			0.17
No	Ref.	Ref.	
Yes	1.00 (0.75–1.35)	0.82 (0.65–1.02)	
Diabetes			< 0.01
No	Ref.	Ref.	
Yes	0.41 (0.18–0.93)	0.25 (0.13–0.46)	
Bleeding in 3rd trimester			0.69
No	Ref.	Ref.	
Yes	0.90 (0.63–1.29)	0.89 (0.67–1.18)	
Anemia			0.13
No	Ref.	Ref.	
Yes	0.98 (0.79–1.21)	1.17 (0.99–1.38)	

Abbreviations: CI, confidence interval; OR, odds ratio.

* Reference category: adequate birth interval.

The variables skin color, age, education level, economic status, type of delivery, number of prenatal visits, number of births, high blood pressure, diabetes, and anemia, whose *p*-value was < 0.2 in the univariate analysis, proceeded to the final model. In the adjusted analysis, only the variable ≥ 3 births (OR= 1.29; CI: 1.01–1.65) was associated with short intervals. Age < 20 years old (OR = 0.48; CI: 0.02–0.12), ≥ 6 prenatal visits (OR = 0.58; CI: 0.47–0.72), ≥ 3 births (OR = 0.59; CI: 0.49–0.73), and gestational diabetes (OR = 0.38; CI: 0.20–0.75) were inversely associated with long interval independently. Age ≥ 35 years old (OR = 2.43; CI: 1.82–3.25) was a factor associated with long birth intervals (Table 4).

**Table 4:** Multivariate analysis of short and long birth intervals, socioeconomic and reproductive health variables, and maternal morbidity

Variables	Short interval*	Long interval*
	OR (95%CI)	OR (95%CI)
Skin color		
White	Ref.	Ref.
Nonwhite	1.39 (1.00–1.95)	1.02 (0.80–1.30)
Age (years old)		
< 20	0.84 (0.56–1.28)	0.48 (0.02–0.12)
20–34	Ref.	Ref.
≥ 35	1.00 (0.65–1.55)	2.43 (1.82–3.25)
Education level (years)		
< 9	Ref.	Ref.
≥ 9	0.83 (0.64–1.07)	0.84 (0.67–1.05)
Economic status		
A–B	Ref.	Ref.
C	0.91 (0.62–1.33)	1.25 (0.95–1.65)
D–E	0.83 (0.54–1.26)	0.82 (0.59–1.14)
Type of delivery		
Normal	Ref.	Ref.
Cesarean section	1.20 (0.93–1.54)	0.95 (0.77–1.17)
Number of prenatal visits		
< 6	Ref.	Ref.
≥ 6	1.21 (0.92–1.60)	0.58 (0.47–0.72)
Number of births		
2	Ref.	Ref.
≥ 3	1.29 (1.01–1.65)	0.59 (0.49–0.73)
High blood pressure		
No	Ref.	Ref.
Yes	0.93 (0.68–1.28)	0.89 (0.69–1.14)
Diabetes		
No	Ref.	Ref.
Yes	0.44 (0.19–1.03)	0.38 (0.20–0.75)
Anemia		
No	Ref.	Ref.
Yes	0.99 (0.79–1.25)	0.99 (0.83–1.20)

Abbreviations: CI, confidence interval; OR, odds ratio.

* Reference category: adequate birth interval.

## Discussion

Birth intervals were predominantly adequate. However, approximately one third of the participating women had long birth intervals. Only the variable ≥ 3 births was associated with short intervals. Age < 20 years old, ≥ 6 prenatal visits, ≥ 3 births, and gestational diabetes decreased the likelihood of long intervals. Age up to 35 years old was associated with long intervals.

Maternal morbidities were determined based on the self-reports of the mothers and on maternity records, when available, without considering their medical records, health professionals' reports, or laboratory tests. This may be considered a limitation of the present study and is likely to have influenced the true prevalence of morbidities across the sample. Furthermore, mothers were considered to have high blood pressure if they reported being diagnosed with the condition during pregnancy, without making any distinction for different types of hypertension (chronic high blood pressure, preeclampsia, and gestational hypertension, etc.).^21^ We point out that the BRISA Study is a population-based research with a large sample size. In addition, as previously mentioned, this survey was conducted in maternity facilities in São Luís and may have peculiar results due to the profile of the population.

The predominance of long intervals was found by other studies, which showed that the prevalence was higher for birth or pregnancy-spacing intervals of > 5 years.^9^ ^12^ ^21^ ^22^ ^23^ The increased intervals between pregnancies may have been caused by the implementation of public policies on family planning in the last decades and an increase in the autonomy of the women over their own reproductive health.^24^

The univariate and multivariate analyses revealed a statistically significant association between the number of prenatal visits and birth intervals, whereby mothers who had at least 6 visits had 58% less chance of having long birth intervals. A study conducted with Arab women in 2012 showed that the number of prenatal visits during the 1^st^ trimester of pregnancy was lower among mothers with short birth intervals.^25^ Similarly, women who had inadequate prenatal care were more likely to have a short subsequent birth interval than those who had adequate care.^13^ The higher contact with health professionals enables health educational actions, which promote healthier attitudes and improvements in family planning. It is worth highlighting that adequate prenatal care is associated with more favorable outcomes in maternal and infant health, especially when the prenatal care group included a multidisciplinary team.^26^ ^27^

The multivariate analysis also showed statistically significant association between age and long birth intervals. Age < 20 years old decreases the chances of long birth intervals, whereas age ≥ 35 years old increases this chance by 43%. The relationship between age and parity is well described in the literature. Often, women with more children are older. Women who have had ≥ 3 births had 29% more chance of having short birth intervals. The negative impact of short birth intervals may only occur in high parity births, usually in older women. This finding is consistent with the concept of maternal depletion as the underlying cause of increased adverse child outcomes.^28^

The univariate analysis showed a statistically significant association between diabetes and birth interval. Having gestational diabetes proved to be a factor associated with long and short birth intervals. After adjustment for socioeconomic variables, age, and parity, the association with long intervals was maintained. A systematic review of the effects of birth interval on women's health highlighted a cross-sectional study undertaken in Latin America that did not find a significant association between diabetes and birth interval.^2^ ^11^ After adjustment for age, number of prenatal visits, and economic status, the association between diabetes and short birth interval was not maintained. In an updated systematic review, there was evidence that short birth intervals were associated with increased risks of subsequent gestational diabetes, but not long intervals.^29^ In the BRISA study, having gestational diabetes was inversely associated with long intervals when compared with adequate birth intervals. A possible explanation for this finding is the potential of prenatal care in monitoring gestational morbidities.^26^

No association was found between prevalence of anemia during pregnancy and birth interval. Despite the fact that anemia is common during pregnancy, the findings in the literature are inconclusive.^2^ ^11^ ^25^ ^30^ Studies conducted in Latin America reported a 30% increase in the risk of anemia among women with birth intervals of < 6 months, while a study undertaken in Nigeria found an increased risk of anemia in birth intervals of < 2 years. However, studies conducted in Bangladesh and Singapore did not find a statistically significant association.^2^ ^11^ ^25^ ^30^ Supporting our findings, the same aforementioned updated review pointed that no study reported outcomes related to maternal anemia and short birth intervals.^29^

There is emerging evidence that women with long birth intervals are at increased risk for labor dystocia, and that short intervals are associated with increased risks of uterine rupture in women attempting vaginal birth after previous cesarean delivery and uteroplacental bleeding disorders (placental abruption and placenta previa).^2^ A recent systematic review including 15 studies shows that birth intervals longer than 18 months were related to decreased risk of maternal morbidity and failed vaginal delivery after previous cesarean section.^31^

The high number of women included in the present study and the recruitment strategy increased the possibility of generalization of the findings for women who reside in capitals in the northeastern region of Brazil with similar characteristics.

Recent studies indicate the importance of identifying factors associated with birth intervals due to different patterns of association shown for preterm birth compared with maternal outcomes. This suggests that increasing maternal age may have discordant effects on associations between short birth intervals and adverse perinatal and maternal outcomes.^32^

## Conclusion

Most of the women in the sample were nonwhite, from economic group C, aged between 20 and 24 years old, had at least 9 years of education, had 2 lifetime births, had natural births, and had at least 6 prenatal care visits. With respect to maternal morbidity, 15.6% of the mothers had high blood pressure, 2.2% had diabetes, 49.5% had anemia, and 9.4% had experienced at least one bleeding episode in the 3^rd^ trimester of pregnancy. The prevalence of adequate birth intervals was higher than that of long and short intervals. However, approximately one third of the participating women had long intervals. A statistically significant association was maintained between the variable ≥ 3 births and short birth interval with multivariate analysis. In this respect, women who were ≥ 35 years old were more likely to have long birth intervals. Age < 20 years old, ≥ 6 prenatal visits, ≥ 3 births, and gestational diabetes decreased the chances of long birth intervals. Moreover, women with gestational diabetes and adequate prenatal care had higher chances of adequate birth interval, indicating that health assistance during pregnancy is extremely important to encourage adequate intervals between gestations.
